# Spirotetramat resistance in *Myzus persicae* (Sulzer) (Hemiptera: Aphididae) and its association with the presence of the A2666V mutation

**DOI:** 10.1002/ps.7103

**Published:** 2022-08-12

**Authors:** Paul A. Umina, Chris Bass, Anthony van Rooyen, Evatt Chirgwin, Aston L. Arthur, Adam Pym, Jo Mackisack, Andrew Mathews, Lisa Kirkland

**Affiliations:** ^1^ Cesar Australia 95 Albert St Brunswick Victoria Australia; ^2^ School of BioSciences The University of Melbourne Parkville Victoria Australia; ^3^ College of Life and Environmental Sciences, University of Exeter Penryn UK

**Keywords:** peach potato aphid, cross‐resistance, resistance mechanism, genetic diagnostic

## Abstract

**BACKGROUND:**

Chemicals are widely used to protect field crops against aphid pests and aphid‐borne viral diseases. One such species is *Myzus persicae* (Sulzer), a global pest that attacks a broad array of agricultural crops and transmits many economically damaging plant viruses. This species has evolved resistance to a large number of insecticide compounds as a result of widespread and repeated chemical use in many parts of the world. In this study, we investigated the evolution of resistance to a new plant protection product, spirotetramat, following reported chemical control failures.

**RESULTS:**

Our study provides clear phenotypic and genotypic evidence of spirotetramat resistance in populations of *M. persicae* from Australia. We show there is cross‐resistance to other insecticides within the same chemical group, namely spiromesifen and spirodiclofen. We also demonstrate that resistance is associated with the previously reported mutation, A2226V in the target site of spirotetramat, acetyl‐CoA carboxylase. Our genetic analysis found all resistant *M. persicae* populations belong to the same multi‐locus clonal type and carry the A2226V mutation, which appears to be inherited as a dominant trait in this species.

**CONCLUSION:**

Our findings provide new insight into the resistance conferred by A2226V and have implications for the control of *M. persicae* in Australia and worldwide. A diagnostic assay developed in this study should serve as a valuable tool for future resistance monitoring and to support the implementation of pest management strategies. © 2022 The Authors. *Pest Management Science* published by John Wiley & Sons Ltd on behalf of Society of Chemical Industry.

## Graphical abstract

Insecticide resistance in *Myzus persicae* is commonplace in many parts of the world. This now includes resistance to spirotetramat (and other Group 23 compounds), which we show is associated with a mutation in acetyl‐CoA carboxylase. This mutation, A2226V, appears to be inherited as a dominant trait in this species.

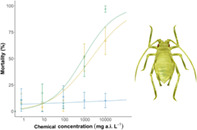

## 1 INTRODUCTION

The green peach aphid or peach potato aphid, *Myzus persicae* (Sulzer), is one of the most damaging aphid pests worldwide. It is highly polyphagous, with a host range of more than 400 species, across 40 different plant families, including many economically important crop plants. Furthermore, *M. persicae* is responsible for the transmission of over 100 plant viruses, such as turnip yellows virus and cucumber mosaic virus.^1^ Globally, control of *M. persicae* has largely relied on the use of synthetic insecticides, and their intensive use over a long period has led to the evolution of resistance to multiple classes of chemistry.^2^ *Myzus persicae* has been confirmed resistant to at least 80 different insecticide compounds, with >470 cases of resistance reported worldwide.^3^ The biochemical and molecular mechanisms of insecticide resistance in *M. persicae* have been extensively studied, with at least eight different resistance mechanisms described to date.^2^, ^4^

In addition to their propensity to evolve resistance, broad host range and ability to transmit plant viruses, the life cycle of *M. persicae* greatly contributes to the pest status of this species globally. Populations of *M. persicae* can undergo both sexual and asexual reproduction, depending on the climate, the availability of its primary winter host (*Prunus* spp.) and the genotypic lineages.^5^ The holocycle of *M. persicae*, with sexual reproduction and overwintering of eggs, occurs in the temperate regions of every continent except Antarctica,^5^ with a mixture of holocyclic (sexual or asexual, host‐alternating) and anholocyclic (asexual, non‐host‐alternating) clones found throughout much of the species’ range. In many countries with a warm climate and/or where *Prunus* spp. are absent, the life cycle is typically anholocyclic. Combined with a short generation time, this mode of reproduction allows *M. persicae* populations to increase rapidly under favourable conditions. Furthermore, if a resistance allele(s) emerges in the field and provides a selective advantage, asexual reproduction enables resistant genotypes to quickly dominate and subsequently spread to new regions.

Similar to many countries around the world, the importance of *M. persicae* as an agricultural pest in Australia has escalated in recent years.^6^ Within Australia, *M. persicae* primarily attacks cucurbit, Solanaceae and brassica vegetable crops, and is a common pest in broadacre grains crops such as canola (oilseed rape) and winter pulses.^7^ *Myzus persicae* feed by sucking sap from plant leaves and flower buds. When population sizes are large, the crop's entire foliage may be covered in aphids, resulting in retarded growth of young plants. Like other aphids, *M. persicae* also secretes honeydew that can result in secondary fungal infection (for example, sooty mould), inhibiting photosynthesis and reducing plant growth and the marketability of the crop.^1^, ^8^ Considerable effort has been made over the last decade to characterise the resistance status of Australian *M. persicae*, with more than 500 field populations collected and screened for resistance between 2012 and 2022^7^, ^9^ (P. A. Umina, unpublished). This work has found widespread resistance to synthetic pyrethroids, organophosphates, carbamates and neonicotinoids across the majority of agricultural regions. More recently, low‐level resistance to the sulfoximine insecticide, sulfoxaflor, has been detected in a small number of field populations of *M. persicae* in the state of Western Australia.^10^ Interestingly, anholocyclic clones that possess multiple resistances dominate Australian agricultural fields^9^ (P. A. Umina, unpublished) and likely undergo parthenogenetic reproduction year round.^11^

Spirotetramat was first registered to control aphid pests in Australia in 2009 and has since become a commonly used aphicide, particularly in vegetable crops. Spirotetramat belongs to the family of tetronic/tetramic acid derivatives or cyclic ketoenols, which are assigned to Group 23 of the Insecticide Resistance Action Committee (IRAC) Mode of Action Classification Scheme.^12^ These compounds are lipid biosynthesis inhibitors targeting acetyl‐CoA carboxylase (ACC), an enzyme known to catalyse the rate‐limiting step in fatty acid biosynthesis.^13^, ^14^ Following uptake by plants, ketoenols are hydrolysed to the active enol form, enabling translocation in the xylem and phloem of crop plants.^14^ It is the enol form that is active against ACC.^14^ In 2020, chemical control failures involving *M. persicae* in a field crop of pepper (*Capsicum frutescens*) were reported near Osborne, Queensland, Australia. The crop was infested with aphids and subsequently sprayed with the recommended label rate of spirotetramat. This spray application failed to achieve adequate control, despite being applied under appropriate weather and spray application conditions. Very recently, population genomic analyses of approximately 130 clones of *M. persicae* collected from around the world identified resistance to spirotetramat in a single clone collected from Australia.^4^ Resistance was associated with the presence of a single non‐synonymous mutation, which results in an alanine to valine substitution (A2226V) in a highly conserved region of the ACC carboxyltransferase domain.^4^ Significantly, although previously undescribed in aphids, the same mutation was recently reported in the whitefly, *Bemisia tabaci* (Gennadius), where its causal role in resistance was confirmed by CRISPR–Cas genome editing.^15^ Despite these findings, the precise level of resistance conferred by this mechanism in *M. persicae*, its cross‐resistance profile in relation to other ketoenol insecticides, its frequency and distribution in Australian *M. persicae* populations, and the implications for control of this species remain unclear.

In this study, the chemical responses of *M. persicae* collected from Osborne were investigated. The sensitivity to spirotetramat in this population was compared with *M. persicae* from several Australian populations, including a known insecticide‐susceptible clone that has been maintained in the laboratory since 2002. After confirming the existence of phenotypic resistance to spirotetramat, we tested several other *M. persicae* populations and demonstrate resistance in multiple populations located in Queensland, Australia. In addition, we show there is cross‐resistance to other ketoenols, namely spiromesifen and spirodiclofen. Genetic analysis confirmed that all resistant populations belong to the same multi‐locus clonal type and carry the A2226V mutation. We also show that spirotetramat resistance has evolved into a genetic background that possesses a large number of other resistance mechanisms. Our findings provide new insight into the resistance conferred by A2226V and have applied implications for the control of *M. persicae* in Australia and worldwide.

## 2 MATERIALS AND METHODS

### 2.1 Aphid collections and culturing

In August 2020, *M. persicae* were collected from a field near Osborne where spirotetramat control failures were reported. Owing to colour variation among individual aphids, we established multiple iso‐female lines from this collection. To do this, individual aphids were transferred to single bok choi (*Brassica napus* subsp. Chinensis) leaves that were placed in 60 mm Petri dishes containing 10 g L^−1^ agar. Petri dishes were kept in a controlled temperature (CT) cabinet set at 18°C, with a 16:8 h light/dark photoperiod. After 9 days, five individuals were removed and placed in 100% ethanol in preparation for clonal assignment via screening of DNA microsatellite markers (Section 2.2). The remaining individuals were transferred to new Petri dishes and maintained under the same conditions described above. This approach of establishing iso‐female lines also allowed us to remove any parasitised aphids and pathogens from the original field collection.^9^ Based on microsatellite markers, two multi‐locus clonal types were identified (herein referred to as Osborne158 and Osborne171). We maintained cultures of each clonal type and tested these separately for their chemical response using bioassays. A known insecticide‐susceptible clone originally collected in 2002 from Kyabram (Victoria, Australia; herein referred to as ‘Kyabram98’) and maintained in the laboratory, was also tested. In addition, a number of other *M. persicae* populations (including some collected nearby to Osborne) were included in bioassays to compare responses. Table 1 provides full details of populations screened to spirotetramat in this study.

**TABLE 1:** Collection details of *Myzus persicae* included in spirotetramat laboratory bioassays

Population	State	Latitude	Longitude	Host plant	Date collected
Kyabram98	Victoria	−36.383	145.033	*Capsicum annum*	1 Apr 2002
Airville188	Queensland	−19.623	147.350	*Capsicum annum*	6 Aug 2013
Elliott158	Queensland	−24.982	152.304	*Capsicum annum*	4 Oct 2017
EastNaernup209	Western Australia	−33.678	120.803	*Brassica napus*	30 Jul 2018
Alloway171	Queensland	−24.955	152.394	*Celosia* sp.	5 Apr 2020
Osborne171^a^	Queensland	−19.706	147.361	*Capsicum frutescens*	26 Aug 2020
Osborne158^a^	Queensland	−19.706	147.361	*Capsicum frutescens*	26 Aug 2020

^a^ Aphids were collected from the same field but contained two different clones.

### 2.2 Identification of aphid clones using microsatellites

Five aphids from each iso‐female line from the Osborne population were genotyped across ten microsatellite loci: M35, M37, M40, M49, M55, M63, M86, myz2, myz9 and myz25^16^ following the DNA extraction and genotyping methods outlined in Umina *et al*.^9^ Loci were labelled with unique fluorophores and co‐amplified in three separate multiplex polymerase chain reactions (PCR) using a Qiagen multiplex kit and an Eppendorf Mastercycler S gradient PCR machine as described in Blacket *et al*.^17^ PCR products were analysed using a 3730 capillary analyser (Applied Biosystems) and genotyping was conducted using GeneMapper version 4.0 (Applied Biosystems).

Additionally, using the same approach described above, we identified the clonal type of aphids from all other *M. persicae* populations phenotypically screened for resistance via laboratory bioassays.

### 2.3 Laboratory bioassays to assess spirotetramat resistance

*Myzus persicae* were screened phenotypically for resistance to spirotetramat using leaf‐dip bioassays, closely following the IRAC Susceptibility Test Method No. 019^18^ and those described in Umina *et al*.^9^ First, we undertook a bioassay comparing the response of *M. persicae* from Kyabram98 to technical grade spirotetramat (>97%, Bayer CropScience) with proprietary formulated product (Movento® 240SC, Bayer CropScience). To make insecticide solutions, technical grade spirotetramat was first dissolved in acetone before diluting in water containing 0.5% Hasten™ adjuvant (Victorian Chemical Company) to create serial dilutions to be tested alongside a control of acetone (plus Hasten™). For Movento® 240SC, we dissolved the chemical product in water containing 0.5% Hasten™ adjuvant to generate a rate of 1000 mg L^−1^, before diluting this stock solution to create serial dilutions to be tested alongside a control of water containing Hasten™. In total, eight concentrations (control [0], 0.001, 0.01, 0.1, 1, 10, 100, 1000 mg L^−1^) were tested for both the technical grade and formulated product. Leaf discs (25 mm diameter) cut from *B. napus* leaves were submerged for 5 s in the insecticide solutions, or the control solutions, and placed adaxial side up on 10 g L^−1^ agar in 35 mm Petri dishes until air‐dry. Six replicate leaf discs were prepared per concentration.

Four days prior to the bioassay, we synchronised the aphid population to ensure all individuals were exposed to spirotetramat at a similar development stage. Approximately 50 apterous aphids were transferred onto *B. napus* leaves placed on 10 g L^−1^ agar within a Petri dish. This Petri dish was placed in a CT cabinet at 18°C with a 16:8 h light/dark photoperiod for 48 h, at which point all adults were removed. The nymphs produced by these adults were retained and returned to the CT cabinet for a further 48 h before being used. Hence, the aphids used in the bioassay were 3–4‐day‐ old nymphs. Approximately eight of these nymphs were transferred to each of the six leaf discs using a fine‐haired paintbrush. After aphid introductions, each Petri dish was inverted onto a lid containing a 25 mm diameter filter paper. Petri dishes were maintained in a CT cabinet held at 18 ± 2°C with a 16:8 h light/dark photoperiod. At 72, 96 and 120 h, aphids were scored as alive (vibrant and moving freely), dead (not moving over a 5 s period) or incapacitated (inhibited movement).^9^

The dose–response curves of aphids tested to technical grade and proprietary formulated spirotetramat were almost identical, particularly after 120 h exposure (*F*_1,81_ = 0.01, *p* = 0.99). Further, median lethal concentration (LC_50_) values were very similar, with overlapping 95% confidence intervals (Figure S1). Based on these findings, a decision was made to conduct all future spirotetramat bioassays using technical grade chemical with an exposure period of 120 h.

**FIGURE 1:**
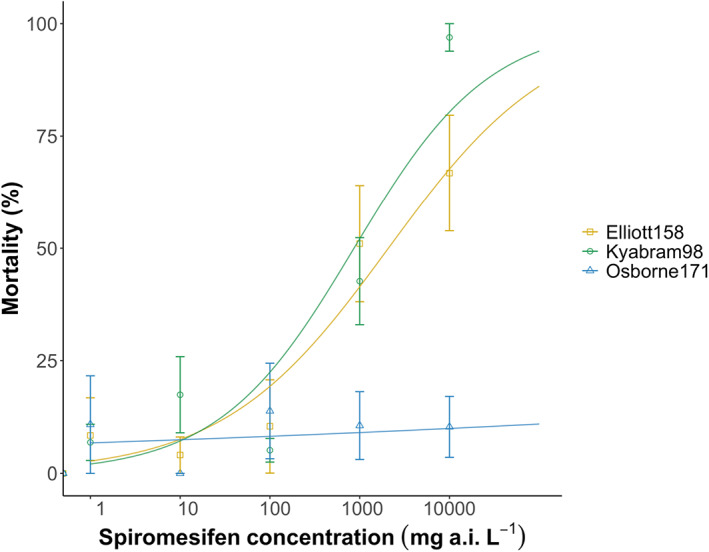
Dose–response curves for *Myzus persicae* when exposed to technical grade spiromesifen for 120 h

A bioassay (bioassay 1) was then conducted comparing the response to spirotetramat of both *M. persicae* clones collected from Osborne (Osborne158 and Osborne171). Following this, we undertook another bioassay (bioassay 2) to examine the responses to spirotetramat of multiple *M. persicae* populations collected between 2017 and 2020 (Table 1). These bioassays followed the methodologies described above and included the insecticide‐susceptible Kyabram98 clone as a control.

### 2.4 Screening for known resistance mechanisms among *M. persicae* populations

Following the detection of phenotypic resistance to spirotetramat (Section 3.1), we leveraged the findings of Singh *et al*.^4^ who recently identified a single non‐synonymous mutation (gCt > gTt) in the heterozygous state at position 2226 in the ACC gene in a single *M. persicae* clone. The Custom TaqMan® Assay Design Tool (https://www.thermofisher.com/order/custom‐genomic‐products/tools/genotyping/) was used to design a TaqMan® SNP Genotyping Assay (ThermoFisher Scientific) for the target site mutation based on sequence information from Singh *et al*.^4^ The assay sequences were forward primer: GGTGAGTTGCGAGGAGGTG, reverse primer: CGACTATCTGGATCTGCATACATCTCAA, reporter sequence VIC (wild‐type): AGTATCTACAACAGCCCATG, reporter sequence FAM (mutant) TAGTATCTACAACAACCCATG. TaqMan® assays were run in duplicate on a LightCycler II 480 real‐time PCR machine (Roche) in a 384‐well format. Each reaction was performed in a 7 μl reaction containing 0.174 μl of the 40× TaqMan® assay, 3.5 μl of 2× KAPA Probe Force qPCR Master Mix (Kapa Biosystems), 1.326 μl of double‐distilled H_2_O and 2 μl of genomic DNA as prepared above. Conditions for the PCR were a pre‐incubation step of 3 min at 98°C followed by 40 cycles of amplification at 95°C for 10 s and 60°C for 20 s, with a final cooling step of 37°C for 1 min. Endpoint genotyping was conducted using the Roche LightCycler 480 software version 1.5.1.62. To confirm the applicability of the assay for discriminating the target site mutation, the assay was tested on six individuals from Kyabram98 and six individuals from the resistant clone that was previously sequenced and identified to carry the A2226V mutation.^4^ These samples were run in duplicate, alongside water (no DNA) controls. Once validated, we applied this diagnostic assay to approximately ten individuals from each of the populations tested phenotypically to spirotetramat (Table 1).

In addition, we screened these same individuals across a number of other resistance mechanisms commonly found in Australian *M. persicae*, including: (i) the acetylcholinesterase enzyme (AChE) mutation S413F, which confers resistance to dimethylcarbamates; (ii) voltage‐gated sodium channel knockdown resistance (*kdr* and *super‐kdr*) mutations L1014F and M918L_ttg, which confer resistance to synthetic pyrethroids; (iii) E4/FE4 esterase genes, which confer resistance to organophosphates; and (iv) increased *CYP6CY3* (P450) copy number, which confers low‐level resistance to neonicotinoids. Although not previously reported in Australia, we also screened for the nicotinic acetylcholine receptor (nAChR) mutation R81T, which is common in regions of Europe and confers high level resistance to neonicotinoids.^19^, ^20^ These assays followed the methods described in Anstead *et al*.,^21^, ^22^ Puinean *et al*.^23^ and Roy *et al*.^24^

To expand our survey of the A2226V mutation, we additionally screened 37 *M. persicae* populations that we had previously collected from agricultural regions across a large part of Australia. This included a mixture of historical and contemporary populations that were sampled from numerous plant hosts. Approximately six individual aphids from each population were screened for the A2226V mutation using the methods described above.

### 2.5 Bioassays to examine cross‐resistance to other ketoenols

To understand the nature of resistance to other Group 23 compounds, we conducted bioassays against spiromesifen (a widely used insecticide to control whiteflies and spider mites) and spirodiclofen (commonly used to control a range of pest mites). The spiromesifen bioassay was conducted in the same manner as that for spirotetramat using a leaf‐dip method (Section 2.3). Technical grade spiromesifen (99.7%, Bayer CropScience) was dissolved in acetone before adding water containing 0.5% Hasten™ and creating serial dilutions to be tested alongside a control of acetone (plus Hasten™). In total, six concentrations (control [0], 1, 10, 100, 1000, 10 000 mg L^−1^) were tested against *M. persicae* from Kyabram98 and Osborne171, along with a third population, Elliott158, which was found to be susceptible to spirotetramat in bioassay 2 (Table 2). Six replicate Petri dishes were prepared per concentration with an average of eight 3–4‐day‐old age‐matched nymphs added per dish. Aphids were kept at 18 ± 2°C with a 16:8 h light/dark photoperiod and mortality was assessed after 120 h as described in Section 2.3.

**TABLE 2:** Summary of *M. persicae* responses to technical grade spirotetramat after 120 h exposure

Bioassay	Population	No. aphids tested	LC_50_ value (mg L^−1^)	Lower‐upper 95% confidence intervals (mg L^−1^)	Regression coefficient (±SE)	Resistance ratio^a^
1	Kyabram98^A^	358	0.095	0.030–0.302	0.644 (0.149)	
	Osborne158^A^	365	0.571	0.199–1.639	0.742 (0.171)	
	**Osborne171** ^**B**^	**390**	**13.121**	**2.887–59.639**	**0.402 (0.090)**	**138.0**
2	Kyabram98^A^	382	0.014	0.004–0.054	0.444 (0.092)	
	EastNaernup209^A^	373	0.017	0.004–0.065	0.429 (0.090)	
	Airville188^A^	387	0.011	0.003–0.046	0.418 (0.089)	
	Elliott158^A^	379	0.031	0.014–0.064	1.057 (0.237)	
	**Alloway171** ^**B**^	**385**	**2.447**	**0.749–7.999**	**0.438 (0.079)**	**170.7**
	**Osborne171** ^**B**^	**377**	**1.515**	**0.495–4.640**	**0.483 (0.088)**	**105.6**

*Note*: Different uppercase letters indicate significant differences between populations within a single bioassay (*p* < 0.05). Populations resistant to spirotetramat are shown in bold.

^a^ Resistance ratios are calculated by dividing the population median lethal concentration (LC_50_) value by the LC_50_ value for Kyabram98.

For spirodiclofen, we tested *M. persicae* from Kyabram98 alongside a clone collected from Bowen (Queensland, Australia; herein referred to as ‘Bowen’) that has previously been shown to have phenotypic resistance to spirotetramat.^4^ Before inclusion of this clone in the bioassay, we extracted DNA from six individual aphids using the Qiagen DNeasy Blood and Tissue kit (Qiagen), with the RNase A treatment step, and then genotyped each aphid across the ten microsatellite loci following the methods described in Section 2.2. We also screened these aphids for the A2226V mutation following the methods described in Section 2.4.

In addition to testing Kyabram98 and Bowen in the spirodiclofen bioassay, we included an insecticide‐susceptible reference clone (4106A), which was collected from pepper in the UK in 2000. As above, we used a leaf‐dip method with 3–4‐day‐old age‐matched nymphs. Bioassays were performed with seven concentrations (control [0], 21, 43, 87, 175, 250, 500 mg L^−1^) of technical grade spirodiclofen (>98%, Merck). Leaf discs (37 mm diameter) cut from *B. napus* leaves were immersed for 10 s in the appropriate concentration of spirodiclofen dissolved in acetone and diluted in 0.02% Triton (Merck). For the controls, leaves were immersed in acetone and 0.02% Triton only. Leaf discs were air‐dried before being placed abaxial side up on 10 g L^−1^ agar in discrete pottles, to which an average of 12 nymphs were added. Four replicate pottles were prepared per concentration. Aphids were then placed in a CT cabinet held at 24 ± 1°C with a 16:8 h light/dark photoperiod and mortality assessed after 72 h.

### 2.6 Bioassays to characterise resistance to other insecticides

To further understand the broader resistance profile of those aphids found to possess spirotetramat resistance, we undertook bioassays against two additional insecticides: sulfoxaflor and flupyradifurone. Sulfoxaflor has been used to control aphids across the globe for more than a decade, with resistance recently discovered in a small number of Australian populations of *M. persicae*.^10^ Flupyradifurone is a newer insecticide, targeting sucking pests^25^ and has only recently been registered against *M. persicae*. Sulfoxaflor bioassays were conducted using a micro‐topical methodology closely following Pym *et al*.^10^ We used a proprietary formulation of sulfoxaflor 240 g L^−1^ (Transform®, Corteva Agriscience), dissolving the chemical product in water to generate a rate of 2400 mg L^−1^, before serially diluting this stock solution in acetone to generate eight test concentrations (control [0], 0.00001, 0.0001, 0.001, 0.01, 0.1, 1, 10 ng of active ingredient [a.i.] per aphid). Six replicates were established for each concentration, with an average of ten adult aphids added to each Petri dish. Each aphid received a volume of 0.25 μl directly onto the prothorax. Aphids were scored as alive, dead or incapacitated after 72 h. We tested three clones, which included Osborne171 and Kyabram98, as well as Munglinup209 (a clone from Western Australia that is known to possess low‐level sulfoxaflor‐resistance, mediated through the overexpression of a P450 gene *CYP380C40* and the UDP‐glucuronosyltransferase gene *UGT344P2*; see Pym *et al*.^10^).

The response of *M. persicae* to flupyradifurone was examined against the same three clones tested against sulfoxaflor. We used a proprietary formulation of flupyradifurone 200 g L^−1^ (Sivanto® Prime, Bayer CropScience), dissolving the chemical product in water to generate a rate of 15 000 mg L^−1^, before serially diluting this stock solution in water to generate the appropriate concentrations. We used a leaf‐dip bioassay, closely following the methodology described above for spirotetramat (Section 2.3). Eight concentrations (control [0], 0.015, 0.15, 1.5, 15, 150, 1500, 15 000 mg L^−1^) were tested on 3–4‐day‐old age‐matched nymphs. Six replicates were used for each concentration, with an average of ten nymphs added to each Petri dish. Aphids were kept at 18 ± 2°C with a 16:8 h light/dark photoperiod and mortality was assessed after 72 h.

### 2.7 Synergism bioassay with piperonyl butoxide

To explore the potential role of metabolic enzymes such as P450s in spirotetramat resistance in *M. persicae*, we conducted a synergism assay using piperonyl butoxide (PBO), which inhibits these enzymes. A preliminary study was conducted to determine the maximum concentration of PBO (diluted in water) that did not cause direct mortality to *M. persicae* (1 g L^−1^, data not shown). Once this was determined, a leaf‐dip bioassay was conducted to examine the responses of the Osborne171 clone and the insecticide‐susceptible Kyabram98 clone when exposed to technical grade spirotetramat alone, or to technical grade spirotetramat following a pre‐treatment with PBO. Pre‐treatments with 1 g L^−1^ PBO were conducted using a Potter Spray Tower (Burkard) approximately 3 h prior to spirotetramat exposure in leaf‐dip assays. The leaf‐dip approach followed the methodology described in Section 2.3. We tested eight concentrations (control [0], 0.001, 0.01, 0.1, 1, 10, 100, 1000 mg L^−1^ prepared in acetone and 0.5% Hasten™). Six replicates were used for each concentration, with an average of eight 3–4‐day‐old age‐matched nymphs added to each Petri dish. Aphids were kept at 18 ± 2°C with a 16:8 h light/dark photoperiod and mortality was assessed after 72 h.

### 2.8 Data analysis

Before data analysis, incapacitated individuals were pooled with dead individuals because they invariably die and therefore do not contribute to the next generation. In addition, we accounted for background mortality by adjusting mortality values at each chemical concentration using Abbott's correction.^26^

Mortality following exposure to each chemical was analysed using logistic regression models, which are well suited for analysing binomial response data (mortality).^27^ To mitigate issues arising from overdispersion (which arose when using a standard binomial distribution), all models used a quasibinomial error distribution.^28^, ^29^, ^30^ For each chemical, we modelled aphid mortality in response to two fixed effect predictors: chemical concentration and aphid population. We subsequently tested whether populations have overall (model intercept) differences in mortality by comparing the change in model deviance (*F* tests). Next, we tested if populations had differences in mortality that were dependent on pesticide concentration (differences in regression slopes) by comparing the change in model deviance when all populations were constrained to have the same intercept (additive predictors only) and when each population had its own slope (the inclusion of population × concentration predictor term). We then used the full model (additive + interactive predictors) to estimate concentrations that resulted in 50% mortality (LC_50_; along with 95% confidence intervals [CI]), calculated using the binomial error distribution. We used these LC_50_ values to estimate the magnitude of insecticide resistance shown by each population compared with the insecticide‐susceptible clone (Kyabram98). Multiple comparisons (post hoc comparisons of intercept and slope between populations) were adjusted with Tukey's HSD method.^31^

For the PBO synergism assay, we used the same modelling approach described above with one additional step to test whether combining spirotetramat + PBO led to higher aphid mortality than the spirotetramat‐only treatment. To do so, we tested whether the model fit improved significantly when our model accounted for PBO compared with a model that ignored PBO using *F* statistics.

All analyses were conducted using R version 3.3.1.^32^

## 3 RESULTS

### 3.1 Detection of field resistance to spirotetramat

Our findings demonstrate that resistance to spirotetramat is present in multiple field populations of *M. persicae* in Australia. The results from bioassay 1 showed significant population differences in response to spirotetramat (*F*_2,122_ = 18.01, *p* < 0.0001), which is further supported by non‐overlapping 95% CIs around the LC_50_ values (Table 2). LC_50_ values were 0.095 and 0.571 mg L^−1^ for Kyabram98 and Osborne158, respectively, whereas Osborne171 had an LC_50_ value of 13.121 mg L^−1^ (Table 2), resulting in a resistance ratio of approximately 138 after 120 h exposure. Osborne171 also had a shallower regression slope compared with Kyabram98 and Osborne158; however, there was no statistically significant difference in slopes between populations (*F*_2,120_ = 2.16, *p* = 0.120).

Results from bioassay 2 further confirmed that Osborne171 is resistant to spirotetramat, while a second population, Alloway171, was also found to possess resistance (Table 2). Strong population differences were again evident (*F*_5,245_ = 14.58, *p* < 0.0001) and there was weak evidence that regression slopes differed between populations (*F*_5,240_ = 2.42, *p* = 0.035). LC_50_ values after 120 h exposure ranged from 0.011 to 0.031 mg L^−1^ for Kyabram98, EastNaernup209, Airville188 and Elliott158, whereas they were 1.515 mg L^−1^ for Osborne171 and 2.447 mg L^−1^ for Alloway171 (Table 2). Resistance ratios were estimated to be approximately 106 and 171 after 120 h exposure for Osborne171 and Alloway171, respectively.

Clonal assignment was performed on all seven populations tested in bioassays 1 and 2 using ten DNA microsatellite markers. This genotyping indicated each population was made up of aphids belonging to a single genetic clonal type per population. It also identified Osborne171 and Alloway171 as the same clonal genotype (Table 3), which was different from all other populations tested. The remaining populations were made up of four distinct multi‐locus clonal types (Table 3).

**TABLE 3:** Clonal make‐up of *Myzus persicae* populations and results from testing of known resistance mechanisms, including A2226V

Population	Clonal type	ACCase (A2226V)	AChE (S431F)	*kdr* (L1014F)	*super*‐*kdr* (M918L)	E4/FE4	nAChR (R81T)	*CYP6CY3*
Alloway171	171	SR	SR	SS	SR	R1	SS	6
Osborne171	171	SR	SR	SS	SR	R1	SS	6
Kyabram98	98	SS	SS	SS	SS	SS	SS	1
Osborne158	158	SS	RR	SS	SR	R2	SS	6
Elliott158	158	SS	RR	SS	SR	R2	SS	6
Airville188	188	SS	SR	SS	SR	R2	SS	3
EastNaernup209	209	SS	RR	SS	SR	R2	SS	3

*Note*: For each resistance loci except *CYP6CY3*, the genotypes of the two alleles are shown with ‘S’ used to denote the susceptible allele and ‘R’ the resistant allele. The copy number of the P450 gene, *CYP6CY3*, is shown, as estimated through quantitative polymerase chain reaction.

### 3.2 Screening of known resistance mechanisms

We screened a number of resistance mechanisms in each of the bioassayed populations, given many of these resistances have been shown to be widespread in Australia. This revealed Osborne171 and Alloway171 are homozygous for the AChE mutation S413F and heterozygous for the *super‐kdr* mutation M918L (Table 3). Osborne171 and Alloway171 were also found to exhibit increased esterase expression when screened for E4/FE4 carboxylesterase and have enhanced expression of *CYP6CY3*. Kyabram98 was confirmed to have no known resistance, while all other populations tested have several known resistance mechanisms. All aphids (including those from Osborne171 and Alloway171) were homozygous wild‐type for the L1014F and R81T mutations (Table 3).

### 3.3 Screening for the A2226V mutation

The new SNP Genotyping Assay designed to detect the A2226V mutation was effective at discriminating control susceptible aphids from resistant aphids that were previously collected from Bowen and shown to carry the single polymorphic mutation.^4^ Additionally, screening *M. persicae* for the A2226V mutation was consistent with our phenotypic bioassay results. Aphids from both Osborne171 and Alloway171 were found to carry one resistant allele at the ACCase locus (i.e., were heterozygous for A2226V), whereas all other aphids, including those from the Kyabram98 susceptible clone, were homozygous susceptible (Table 3).

Following detection of the A2226V mutation in Osborne171 and Alloway171, we undertook widespread screening across 37 additional *M. persicae* populations collected throughout Australia between 2012 and 2021. This revealed an additional seven populations that are heterozygous for the A2226V mutation (Table S1). Genotypic analysis found that each resistant population was made up of aphids belonging to a single genetic clonal type, which is identical to Osborne171 and Alloway171. Interestingly, however, there are populations of the same clonal type that were found to be homozygous wild‐type for A2226V (Table S1).

### 3.4 Cross‐resistance to spiromesifen and spirodiclofen

There were substantial differences in response to spiromesifen between aphids from Osborne171 and those from both Kyabram98 and Elliott158. Very low mortality was observed across all concentrations tested (up to 10 000 mg L^−1^) in the Osborne171 clone (Figure 1 and Table 4), which precluded the estimation of LC_50_ values. This was not the case for aphids from Kyabram98 and Elliott158, which were previously found to be susceptible to spirotetramat (Tables 2 and 4). Still, populations differed significantly in their intercept (*F*_2,86_ = 3.81, *p* < 0.05) and slope (*F*_2,84_ = 3.54, *p* < 0.05). Post hoc tests showed Osborne171 had a significantly smaller intercept (*p* = 0.044) and slope (*p* = 0.031) compared with Kyabram98 (Table 4).

**TABLE 4:** Summary of *Myzus persicae* responses to technical grade spiromesifen after 120 h exposure

Population	No. aphids tested	LC_50_ value (mg L^−1^)	Lower‐upper 95% confidence intervals (mg L^−1^)	Regression coefficient (±SE)	Mortality (%) at 10 000 mg a.i. L^−1^
Kyabram98	267	864.46	245.0–3050.8	0.576 (0.153) ^A^	97.2
Elliott158	288	2088.5	443.7–9831.2	0.471 (0.131) ^AB^	75.0
Osborne171	282	NC^a^		0.046 (0.143) ^B^	14.8

*Note*: Different uppercase letters indicate significant differences between population slopes (regression coefficients) (*p* < 0.05).

^a^ NC indicates populations for which median lethal concentration (LC_50_) values could not be estimated due to low aphid mortality (even at the highest concentrations tested).

Analogous with this finding, we found evidence for cross‐resistance to spirodiclofen in *M. persicae* that were resistant to spirotetramat. Populations exposed to spirodiclofen for 72 h differed significantly in their intercept (*F*_2,148_ = 47.18, *p* < 0.0001) but not their slope (*F*_2,146_ = 2.66, *p* = 0.066). Specifically, the Bowen clone was significantly more resistant than Kyabram98 and a second susceptible clone, 4106A (Table 5). The resistance ratio of the Bowen clone was found to be approximately 114 after 72 h exposure. Genetic screening of aphids from Bowen found an identical clonal profile as aphids from Osborne171 and showed all Bowen individuals were heterozygous for the A2226V mutation.

**TABLE 5:** Summary of *Myzus persicae* responses to technical grade spirodiclofen after 72 h exposure

Population	No. aphids tested	LC_50_ value (mg L^−1^)	Lower‐upper 95% confidence intervals (mg L^−1^)	Regression coefficient (±SE)	Resistance ratio^a^
Kyabram98^A^	318	6.88	1.44–32.94	1.156 (0.481)	
4106A^B^	322	33.69	21.91–51.81	0.827 (0.102)	4.9
Bowen^C^	327	783.17	226.21–2711.40	0.513 (0.101)	113.8

*Note*: Different uppercase letters indicate significant differences between populations (*p* < 0.05).

^a^ Resistance ratios are calculated by dividing the population median lethal concentration (LC_50_) value by the LC_50_ value for Kyabram98.

### 3.5 Resistance to sulfoxaflor and flupyradifurone

We found variable responses when testing *M. persicae* against sulfoxaflor and flupyradifurone. In the case of flupyradifurone, responses for Kyabram98, Osborne171 and Munglinup209 were similar; there was no statistical difference between populations (*F*_2,122_ = 0.97, *p* = 0.383) and LC_50_ values had overlapping 95% CIs (Table 6). There was also no significant difference in regression slopes between populations (*F*_2,120_ = 1.40, *p* = 0.250). This shows there is no cross‐resistance between spirotetramat and flupyradifurone. In the case of sulfoxaflor, there were significant population differences (*F*_2,122_ = 9.21, *p* < 0.001). Munglinup209 was confirmed as possessing resistance, with a resistance ratio of approximately 14, which is consistent with previously published data.^10^ Surprisingly, there was also a significant difference in responses to sulfoxaflor between Kyabram98 and Osborne171. Aphids from Osborne171 were also found to be resistant, with a resistance ratio of approximately 24 (Table 6). This indicates Osborne171 individuals possess resistance to both spirotetramat and sulfoxaflor.

**TABLE 6:** Summary of *Myzus persicae* responses to sulfoxaflor and flupyradifurone after 72 h exposure

Chemical	Population	No. aphids tested	LC_50_ value (mg L^−1^)	Lower‐upper 95% confidence intervals (mg L^−1^)	Regression coefficient (±SE)	Resistance ratio^a^
Sulfoxaflor	Kyabram98^A^	386	0.003	0.001–0.011	0.431 (0.073)	
	Osborne171^B^	484	0.082	0.028–0.234	0.423 (0.066)	24.4
	Munglinup209^B^	430	0.047	0.0167–0.129	0.478 (0.076)	14.0
Flupyradifurone	Kyabram98^A^	431	15.267	8.008–29.106	1.029 (0.192)	
	Osborne171^A^	488	19.314	9.230–40.414	0.710 (0.105)	
	Munglinup209^A^	487	7.878	4.137–15.001	0.924 (0.153)	

*Note*: Different uppercase letters indicate significant differences between populations for each chemical (*p* < 0.05).

^a^ Resistance ratios are calculated by dividing the population median lethal concentration (LC_50_) value by the LC_50_ value for Kyabram98 for sulfoxaflor.

### 3.6 Synergism assays

There was no evidence that pre‐treatment of *M. persicae* with the metabolic enzyme inhibitor PBO impacted the level of resistance to spirotetramat. There was no significant difference when we modelled aphid mortality and accounted for PBO treatment and population compared with a model that only accounted for population (*F*_5,160_ = 1.14, *p* = 0.34). Indeed, Osborne171 showed similar levels of resistance to spirotetramat whether individuals were pre‐treated with PBO or not; LC_50_ values and regression slopes were not statistically significant (Table S2). This suggests P450s are unlikely to play a role in conferring spirotetramat resistance in *M. persicae* and further supports our earlier assertion for the causal role of the A2226V mutation in resistance to ketoenols.

## 4 DISCUSSION

This study provides clear phenotypic and genotypic evidence of spirotetramat resistance in *M. persicae* from Queensland, Australia. Our data provides insight into the resistance phenotype to this insecticide and other ketoenols in *M. persicae*, the underpinning mechanism at play, and the distribution of this new resistance in Australia. In addition, we determine the broader resistance profile of the *M. persicae* clone possessing spirotetramat resistance, finding a long list of other resistances also present. We discuss these topics and their fundamental and applied implications below.

Insecticide bioassays revealed high levels of resistance (approximately 105–170‐fold relative to a reference susceptible clone) to spirotetramat in two populations of *M. persicae* collected from Queensland. This level of resistance is consistent with that reported in *B. tabaci*, where a strain collected from Australia exhibited a resistance ratio of more than 165‐fold to spirotetramat.^15^ Our findings also corroborate the identification of a single spirotetramat‐resistant clone of *M. persicae* from Queensland in our previous analysis of globally collected samples.^4^ In this previous study, resistance was identified based on 100% survival at two discriminating dose concentrations of spirotetramat.^4^ The results presented here provide the first quantitative measure of resistance to spirotetramat in *M. persicae*. Furthermore, we demonstrate that resistance extends to other ketoenol insecticides, namely spiromesifen and spirodiclofen, revealing cross‐resistance within this Mode of Action (MoA) group; once again, consistent with the resistance profile reported for *B. tabaci*.^15^ The levels of ketoenol resistance we have identified are expected to impair the efficacy of spirotetramat when used at the recommended rate (48 g a.i. ha^−1^) against *M. persicae* in the field. Thus, the chemical control failures involving *M. persicae* reported near Osborne, in Queensland (see Introduction) are likely to be a direct result of resistance rather than other factors such as suboptimal application practices.

Previous research on a laboratory‐selected spirotetramat‐resistant strain of the cotton aphid, *Aphis gossypii* (Glover), found that the P450 inhibitor PBO significantly increased the toxicity of spirotetramat in the resistant strain.^33^ Subsequent molecular and functional analyses implicated overexpression of the P450 *CYP6A2* in resistance. However, in the current study, no significant synergism was observed when PBO was used in spirotetramat bioassays, suggesting P450s are unlikely to play a major role in resistance in *M. persicae*. Rather, we show that resistance is associated with the previously reported mutation, A2226V in the target site of spirotetramat, ACC.^4^ This mutation occurs in a highly conserved region of the ACC carboxyltransferase domain, and its causal role in resistance has been demonstrated by introducing the mutation into *Drosophila melanogaster* by genome editing.^15^ All resistant aphids identified in our study carried the mutation in the heterozygous form, consistent with the initial report of this mutation in *M. persicae*.^4^ This finding, in combination with our phenotypic analysis of clones possessing the mutation, suggests that A2226V is inherited as a dominant trait (heterozygous individuals are resistant) in *M. persicae*, consistent with the autosomal dominant mode of inheritance reported in *B. tabaci*.^15^ Our results also strongly suggest that A2226V confers resistance to a range of ketoenols, in agreement with the cross‐resistance profile of this mutation in *B. tabaci*.^15^

Our widespread screening of 37 *M. persicae* populations collected throughout Australia, sampled between 2012 and 2021, revealed a further seven populations that are heterozygous for A2226V. Interestingly, all seven populations originate from a relatively small region of Queensland, which is also where both Osborne171 and Alloway171 aphids were collected. This region of Queensland has high‐intensity production of fruit and vegetable crops, such as tomatoes, peppers, pumpkins and eggplants. Intriguingly, the first report of spirotetramat resistance, and the A2226V mutation, in Australian *B. tabaci* was also found in populations collected from this area of Queensland.^15^ Further research may be warranted to explore whether high insecticide selection pressure in this region makes it a ‘hot spot’ for resistance evolution. In addition to the spatial distribution of resistance, our data show the A2226V mutation has been present in Australian *M. persicae* since at least 2013 and is likely to have persisted in the field since that time, given that several populations collected in both 2013 and 2021 have been found to carry the mutation. Given that spirotetramat has been registered to control aphid pests in Australia since 2009, this finding reveals that resistance likely evolved within just 4 years of spirotetramat use. This, once again, illustrates the remarkable evolutionary capacity of *M. persicae* to rapidly adapt to insecticide selection.^2^

Genotypic analysis found that each *M. persicae* population carrying A2226V was made up of aphids belonging to a single genetic clonal type (clone 171), which is identical to the clone from Bowen, Queensland, in which the A2226V mutation was first identified. These results are consistent with a single de novo origin of resistance occurring in a single lineage of *M. persicae* in Australia. The fact that the A2226V mutation is inherited as a dominant trait in this species means an immediate fitness benefit would be experienced by aphids possessing this resistance in the presence of ketoenol insecticides, favouring its establishment and spread. Anholocyclic clones of *M. persicae* that undergo parthenogenetic reproduction year round have been shown to dominate within Australia,^9^, ^11^ meaning de novo resistance mutations, and combinations of mutations, can become ‘locked’ in clonal lineages. This situation likely explains why only a single genotypic lineage has been found to carry A2226V (and always in the heterozygous form). Interestingly, however, our genotypic analysis found that several *M. persicae* populations in Australia identified as clone 171 do not possess the A2226V mutation. This is likely to be the result of this mutation evolving in a parthenogenetic clonal type, although other contributing factors (for example, recombination) cannot be excluded.^34^ Regardless, there now exists a spirotetramat‐resistant clone that is indistinguishable from non‐resistant clones when using previously deployed DNA microsatellite markers. It will be important to develop more‐sensitive approaches to identify genetic clones if attempting to infer the resistance status of field populations from clonal type alone. One such approach that has greater power and has been used successfully in other pest management contexts is a double digest RAD sequencing approach to analyse genome‐wide single nucleotide polymorphism variation.^35^, ^36^

In anholocyclic lineages of *M. persicae* the whole genome is effectively in complete linkage and it is thus notable that we found the A2226V mutation in a genetic background containing multiple resistance mechanisms to other insecticides, including the AChE mutation S413F, conferring resistance to carbamates; the *super‐kdr* mutation M918L, conferring resistance to pyrethroids; increased esterase expression, indicating resistance to organophosphates; and enhanced expression of *CYP6CY3*, conferring resistance to neonicotinoids. Our phenotypic bioassays also found this clone to be resistant to sulfoxaflor. Thus, these aphids possess resistance to insecticides belonging to at least six MoA subgroups. To our knowledge, no other *M. persicae* clone has been found with this number of resistance mechanisms globally. This clone is, therefore, expected to be a major challenge for growers when controlling aphids with insecticides, although we found no evidence of cross‐resistance to flupyradifurone in this study. In a separate study, we tested the responses of *M. persicae* (including Osborne171 and Kyabram98) against two other insecticides recently registered in Australia, flonicamid (MoA Group 4D) and afidopyropen (MoA Group 9D).^37^ We found no differences between the sensitivities of Osborne171 and Kyabram98 to either insecticide, suggesting a lack of cross‐resistance between spirotetramat resistance and these MoA groups. Combined, these results highlight the opportunity to use flupyradifurone, flonicamid and afidopyropen to manage Australian populations of *M. persicae* in the future.

In conclusion, we provide new insight into the important role of A2226V in conferring resistance to spirotetramat in *M. persicae* and the resistance phenotype conferred by this mechanism in this species. Given the current restricted distribution of this mechanism in a single Australian state it is imperative to conduct further regular monitoring of *M. persicae* populations in Australia and worldwide. In this regard, the diagnostic assay developed in this study provides a powerful tool for future resistance monitoring. Equally important will be the implementation of management strategies to delay the spread of spirotetramat resistance, and/or additional de novo origins of the same mutation (as has occurred in *B. tabaci*).^15^ These strategies should include the adoption of non‐chemical control options and the strategic rotation of those insecticides that remain effective against *M. persicae*.

## Supporting information


**Appendix S1.** Supporting information.

## Data Availability

The data that support the findings of this study are available from the corresponding author upon reasonable request.
