# Supplementary material for: Spirotetramat resistance in Myzus persicae (Sulzer) (Hemiptera: Aphididae) and its association with the presence of the A2666V mutation
Source: Pest Manag Sci. 2022 Aug 12;78(11):4822–31. doi: 10.1002/ps.7103 (PMC9804573; doi:10.1002/ps.7103)
Supplement: Supplementary file 1 — Appendix S1. Supporting information. [file PS-78-4822-s001.docx]

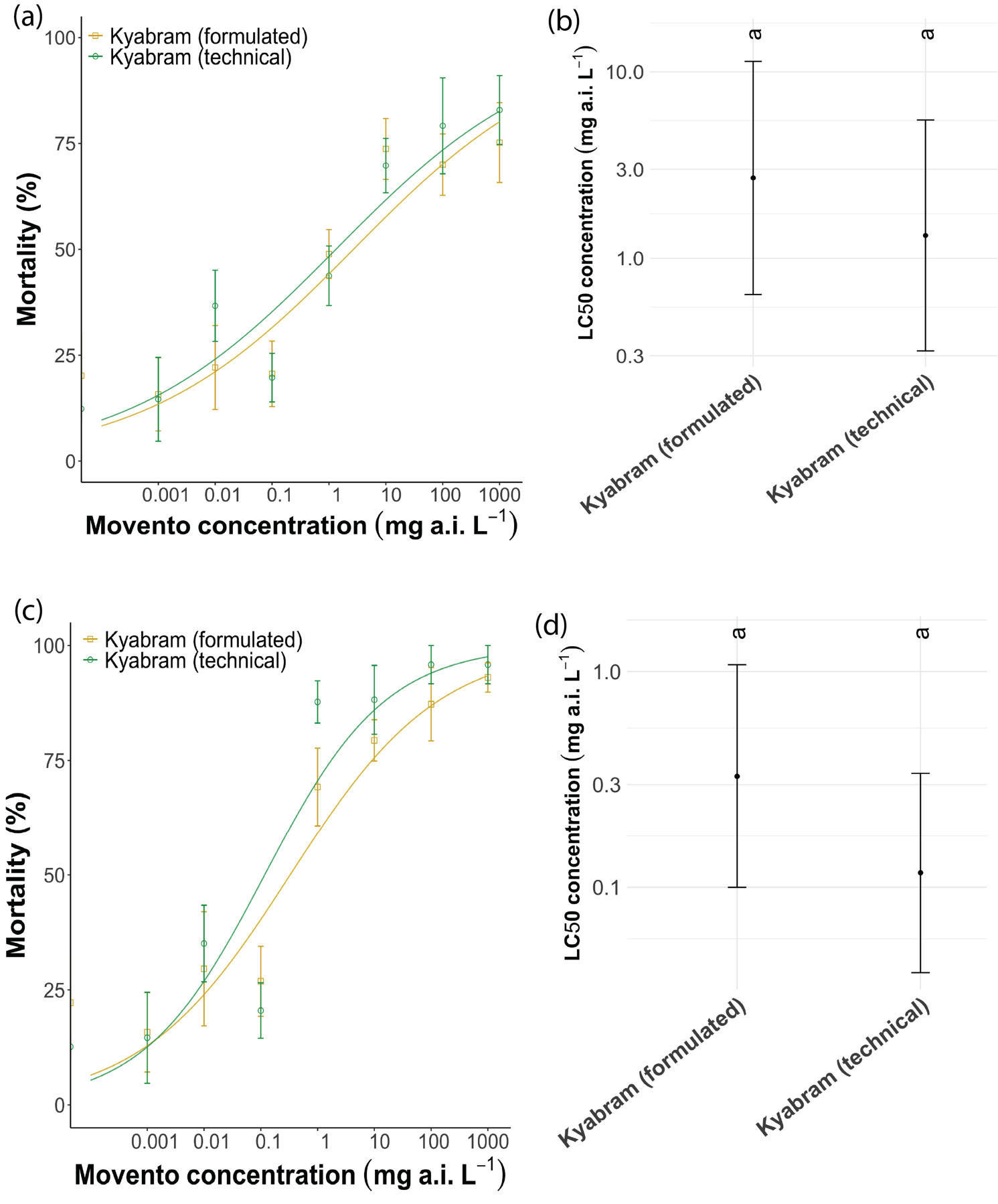


**
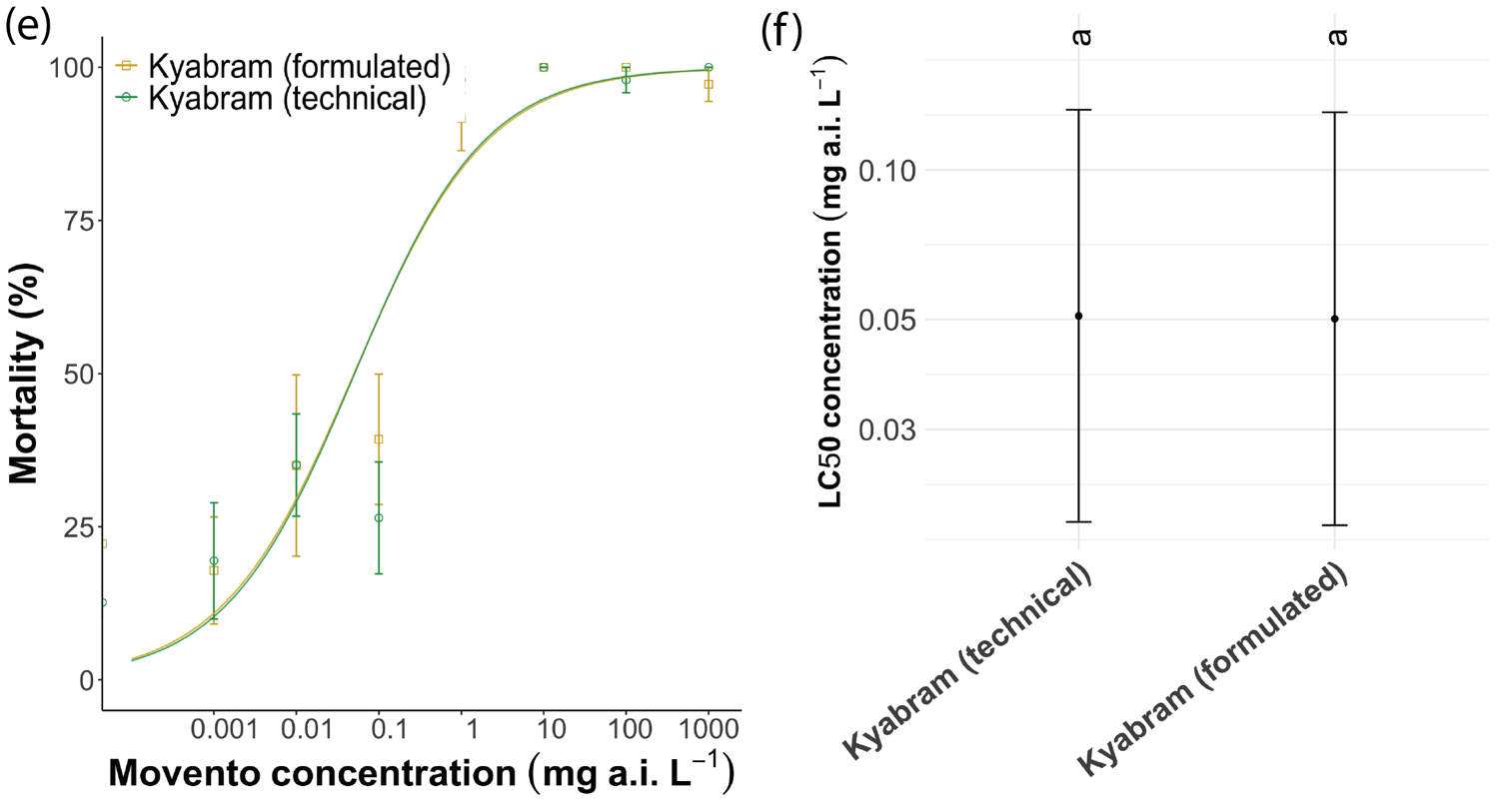
**

**Suppl. Fig. 1.** Comparison of technical grade and formulated product of spirotetramat when tested against an insecticide-susceptible *M. persicae* clone (Kyabram98). (a) shows dose-response curves after 72 h exposure, (b) shows LC_50_ values (and 95% confidence intervals) after 72 h exposure, (c) shows dose-response curves after 96 h exposure, (d) shows LC_50_ values (and 95% confidence intervals) after 96 h exposure, (e) shows dose-response curves after 120 h exposure, and (f) shows LC_50_ values (and 95% confidence intervals) after 120 h exposure.

**Suppl. Table 1.** Widespread screening of the A2226V mutation within Australian populations of *M. persicae.* Populations are ordered by latitude from north to south. Populations found with the A2226V mutation are shown in bold.

| **Population** | **State** | **Latitude** | **Longitude** | **Host plant** | **Date collected** | **Clonal type** | **A2226V** |
| --- | --- | --- | --- | --- | --- | --- | --- |
| **Colevale171** | **Queensland** | **-19.505** | **147.328** | ***Capsicum chinense*** | **27 Aug 2021** | **171** | **SR** |
| **Ayr171** | **Queensland** | **-19.583** | **147.400** | ***Capsicum annum*** | **19 Aug 2013** | **171** | **SR** |
| **Mt Kelly171** | **Queensland** | **-19.696** | **147.319** | ***Cleome aculeata*** | **10 Aug 2021** | **171** | **SR** |
| Fredericksfield22 | Queensland | -19.717 | 147.378 | *Capsicum annum* | 09 Aug 2013 | 22 | SS |
| **Wunjunga171** | **Queensland** | **-19.839** | **147.608** | ***Capsicum annum*** | **06 Aug 2013** | **171** | **SR** |
| Guthalungra158 | Queensland | -19.864 | 147.670 | *Capsicum annum* | 22 June 2016 | 158 | SS |
| **GumluSouth171** | **Queensland** | **-19.892** | **147.689** | ***Capsicum annum*** | **06 Aug 2013** | **171** | **SR** |
| **BowenNorth171** | **Queensland** | **-19.978** | **148.193** | ***Capsicum annum*** | **15 Oct 2013** | **171** | **SR** |
| Bowen158 | Queensland | -20.010 | 148.188 | *Solanum melongena* | 16 Aug 2021 | 158 | SS |
| **BowenEast171** | **Queensland** | **-20.052** | **148.145** | ***Capsicum annum*** | **15 Oct 2013** | **171** | **SR** |
| MtMurchison171 | Queensland | -24.374 | 150.577 | *Brassica tournefortii* | 04 May 2012 | 171 | SS |
| MooreParkBeach209 | Queensland | -24.737 | 152.299 | *Solanum melongena* | 16 Oct 2013 | 209 | SS |
| Barakula51 | Queensland | -26.440 | 150.370 | *Brassica oleracea* | 18 June 2012 | 51 | SS |
| Pittsworth209 | Queensland | -27.716 | 151.633 | *Brassica oleracea* | 13 April 2017 | 209 | SS |
| SpringCreek209 | Queensland | -27.933 | 151.966 | *Brassica oleracea* | 26 Mar 2012 | 209 | SS |
| Allenview171 | Queensland | -27.934 | 152.917 | *Brassica rapa* | 21 Mar 2012 | 171 | SS |
| Northampton209 | Western Australia | -28.351 | 114.628 | *Brassica napus* | 03 Aug 2016 | 209 | SS |
| SouthGreenough171 | Western Australia | -29.028 | 114.832 | *Capsicum annum* | 14 June 2016 | 171 | SS |
| Eganu209 | Western Australia | -30.025 | 115.763 | *Brassica napus* | 15 Sept 2020 | 209 | SS |
| Mocardy209 | Western Australia | -30.800 | 116.923 | *Brassica napus* | 28 June 2017 | 209 | SS |
| EbaAnchorage209 | South Australia | -32.722 | 134.295 | *Brassica napus* | 30 July 2014 | 209 | SS |
| Cascade209 | Western Australia | -33.474 | 120.903 | *Brassica napus* | 15 Aug 2018 | 209 | SS |
| Munglinup209 | Western Australia | -33.681 | 120.820 | *Brassica napus* | 29 Aug 2018 | 209 | SS |
| Nericon209 | New South Wales | -34.207 | 146.081 | *Brassica napus* | 16 July 2017 | 209 | SS |
| StockyardCreek183 | South Australia | -34.288 | 138.596 | *Brassica napus* | 10 Sept 2018 | 183 | SS |
| Virginia171 | South Australia | -34.650 | 138.557 | *Capsicum annum* | 14 Aug 2014 | 171 | SS |
| Dirnaseer209 | New South Wales | -34.660 | 147.819 | *Brassica napus* | 06 Oct 2017 | 209 | SS |
| Wanbi217 | South Australia | -34.761 | 140.345 | *Brassica napus* | 01 Sept 2017 | 217 | SS |
| Picola171 | Victoria | -35.998 | 145.097 | *Brassica napus* | 21 June 2017 | 171 | SS |
| Albury209 | New South Wales | -36.073 | 146.913 | *Brassica napus* | 17 July 2017 | 209 | SS |
| Nhill209 | Victoria | -36.332 | 141.650 | *Brassica napus* | 14 July 2017 | 209 | SS |
| Rochester209 | Victoria | -36.357 | 144.662 | *Brassica napus* | 21 June 2017 | 209 | SS |
| Cosgrove171 | Victoria | -36.376 | 145.595 | *Brassica napus* | 28 Sept 2016 | 171 | SS |
| Markwood171 | Victoria | -36.456 | 146.490 | *Brassica napus* | 27 April 2016 | 171 | SS |
| WhoroulyEast171 | Victoria | -36.534 | 146.660 | *Brassica rapa* | 18 May 2016 | 171 | SS |
| MurchisonNorth171 | Victoria | -36.591 | 145.243 | *Antirrhinum majus* | 17 Sept 2013 | 171 | SS |
| Winchelsea209 | Victoria | -38.178 | 144.001 | *Brassica napus* | 08 May 2017 | 209 | SS |

**Suppl. Table 2.** Summary of *M. persicae* responses to technical grade spirotetramat with and without the synergist PBO after 120 h exposure.

| **Population** | **Synergist** | **No. aphids tested** | **LC_50_ value (mg L^-1^)** | **Lower-upper 95% confidence intervals (mg L^-1^)** | **Regression coefficient (±SE)** |
| --- | --- | --- | --- | --- | --- |
| Kyabram98 ^A^ | Nil | 376 | 0.005 | 0.002 – 0.014 | 0.534 (0.103) |
| Kyabram98 ^A^ | PBO | 363 | 0.002 | 0.0004-0.010 | 0.389 (0.083) |
| Osborne171 ^B^ | Nil | 383 | 2.093 | 0.944 – 4.645 | 0.641 (0.098) |
| Osborne171 ^B^ | PBO | 375 | 1.053 | 0.451 – 2.462 | 0.570 (0.086) |

*Note:* Different uppercase letters indicate significant differences between populations (*p* < 0.05).
